# Beyond initial adoption: addressing the know-do gap in technology-enhanced medical education

**DOI:** 10.1080/10872981.2026.2694914

**Published:** 2026-06-25

**Authors:** Rui Wang, Xiaoshuai Li

**Affiliations:** a Department of Stem Cells and Regenerative Medicine, Key Laboratory of Cell Biology, Ministry of Public Health, Key Laboratory of Medical Cell. Biology, Ministry of Education, China Medical University, Shenyang, Liaoning, People's Republic of China; b Department of Blood Transfusion, Shengjing Hospital of China Medical University, Shenyang, Liaoning, People's Republic of China; c NHC Key Laboratory of Congenital Malformation, Shengjing Hospital of China Medical University, Shenyang, Liaoning, People's Republic of China

**Keywords:** Faculty development, Universal design for learning, Web 2.0 tools, technology adoption, medical education

To the Editor,

I read with great interest the study by Ergönül and Büyükçoban evaluating a faculty development programme that integrates Web 2.0 tools with Universal Design for Learning (UDL) principles in medical education [1]. As a medical educator, I commend the authors for tackling one of the most persistent challenges in faculty development: translating andragogical training into sustained classroom practices. Their finding that none of the 262 participants had prior familiarity with UDL, coupled with the statistically significant increases in UDL adoption and Web 2.0 tool usage post-training, provides encouraging evidence that even brief experiential interventions can shift educator awareness. The study's mixed-methods design further enriches these findings by revealing the complex interplay of power dynamics, emotional labour, and institutional resistance underlying technology adoption.

However, I would like to draw attention to a finding that the authors acknowledge yet, in my view, deserves deeper interrogation as the central puzzle of their results: while statistically significant increases in tool usage were observed across all nine Web 2.0 tools, absolute adoption rates two months post-training remained modest. Wooclap, a tool explicitly designed to enhance student engagement, the very outcome the programme prioritised, was frequently used by only 29.5% of participants, while tools such as Mindomo, Edpuzzle, and Thinglink showed negligible adoption. This pattern reveals what might be termed a know-do gap: participants learned to use these tools and recognised their andragogical value, yet the majority did not integrate them into practice.

The authors interpret this gap primarily through the lens of institutional barriers, such as time constraints, workload, and lack of technical support. While these factors are undoubtedly critical, I would argue that the qualitative themes identified in this study point toward an equally important but less explored explanation: andragogical identity disruption.

The qualitative findings of this study are extraordinarily revealing in this respect. Theme 1—‘Shifting Power Dynamics and Andragogical Vulnerability’—captures educators' anxiety about losing classroom authority when technology redistributes participation. The participant who stated, ‘I need to be ready for the answers provided’ was articulating a fundamental renegotiation of professional identity, from knowledge transmitter to learning facilitator. Theme 3—‘Reaction to Institutional Constraints: Resistance and Technostress’—reveals that ‘I don't have time’ may function, as the authors perceptively note, as ‘a coping strategy to safeguard professional space and academic confidence.’ This ‘vulnerability of the expert’ is particularly acute in medical education, where a culture of perfectionism can render the unpredictability of live interactive feedback a perceived threat to professional credibility. Non-adoption may then represent not a failure of training but a rational protective response to threats to professional identity.

The literature on teacher change supports this interpretation of the results. Meaningful andragogical transformation requires what Ertmer and Ottenbreit-Leftwich term ‘deep belief change’—a reconceptualization of what it means to be an effective educator [2]. Guskey's model of teacher change further posits that significant shifts in teacher beliefs occur only after educators observe positive student learning outcomes resulting from new practices—a sequence requiring considerably longer than the two-month evaluation window employed in this study [3]. Educators gravitated toward ‘safe’ tools such as Google Forms (50% adoption) while avoiding more ‘disruptive’ yet engagement-centric tools such as Wooclap (29.5%); this is fully consistent with this framework: early adoption favours tools that minimise identity threat.

Therefore, I propose three shifts in how we approach faculty development for technology integration: **1. The evaluation window should be extended.** Future studies should incorporate follow-up assessments at six and 12 months to capture the trajectory from initial adoption to sustained integration. Late adoption, driven by positive student feedback reinforcing the value of new practices, may be missed by short-term evaluations. **2. Designing for identity works alongside designing for skills.** Faculty development programmes should explicitly address the emotional and identity-related dimensions of andragogical change. Creating structured peer observation networks, safe-fail practice opportunities, and facilitated reflection on professional identity may help educators navigate the vulnerability that the authors perceptively identify. Institutions must foster a culture of psychological safety that rewards andragogical experimentation and explicitly validates the trial-and-error phase inherent in adopting interactive andragogies. **3. Differentiating institutional support by adoption phase.** Educators in the early adoption stage need psychological safety and identity support (mentoring, peer communities), while those progressing toward sustained integration need logistical support (reduced workload, technical assistance). A one-size-fits-all institutional response misses the distinct needs of different phases of the adoption journey.

Finally, it is worth situating this discussion within the broader context of medical education. As generative AI reshapes knowledge acquisition, the teacher's role is shifting inexorably from ‘content deliverer’ to ‘human-centric facilitator.’ Beyond andragogy, the emerging paradigm of heutagogy—self-determined learning—calls on educators to cede control so that learners can define their own learning questions, methods, and assessments [4]. Viewed in this light, the UDL framework provided by Ergönül and Büyükçoban represents not merely a tool for enhancing interactivity but a foundational competency for educators navigating an AI-augmented educational landscape.

Ergönül and Büyükçoban have provided the field with a valuable model and unusually candid qualitative data illuminating the inner experience of educators navigating this transition. The know-do gap their data reveal is not a weakness of their intervention but a window into the deeper processes of professional change. Addressing these identity-level dynamics alongside institutional structures may be key to converting promising initial adoption into a sustained, transformative andragogical practice.

## Data Availability

Reported as not applicable.

## Previous presentations

None declared.
